# Relationship between faecal egg count and health status in Nguni goats reared on semi-arid rangelands

**DOI:** 10.1007/s11250-023-03483-w

**Published:** 2023-03-30

**Authors:** S. Z. Ndlela, M. V. Mkwanazi, M. Chimonyo

**Affiliations:** 1 Animal and Poultry Science, School of Agricultural, Earth and Environmental Sciences, University of KwaZulu-Natal, P Bag X01 Scottsville, Pietermaritzburg, 3209 South Africa; 2 Faculty of Science, Engineering and Agriculture, University of Venda, P Box X5050, Thohoyandou, Thohoyandou, South Africa

**Keywords:** FAMACHA, Nematodes, Packed cell volume, Seasonal prevalence

## Abstract

Gastrointestinal parasitism is a major constraint to goat productivity, particularly in resource-limited production systems. The objective of the study was to determine the relationship between faecal egg count and the health status of different classes of Nguni goats. Body condition score (BCS), packed cell volume (PCV), FAMACHA score, and faecal egg count (FEC) were measured in 120 goats of different classes (weaners, does and bucks) across seasons. The identified gastrointestinal nematodes (GIN) were *Strongyloides* (30 %), *Haemonchus contortus* (28 %), *Trichostrongylus* sp. (23 %), *Oesophagostomum* sp. (17 %) and *Ostertagia* (2 %), which showed higher prevalence at the hot-wet season compared to other seasons. An interaction (*P*<0.05) between class and season on BCS was observed. Lower PCV were observed in weaners (24.6 ± 0.79) in the post-rainy season, whereas does 27.4 ± 0.86 and bucks (29.3±1.03) had the highest PCV in the same season. Higher FAMACHA scores were observed in the hot seasons for all goat classes, while lower in the cool-dry season. Linear relationships between FAMACHA scores and FEC were observed in all seasons. The rate of change in FAMACHA score was higher in the post-rainy season (*P*<0.01) than in other seasons as FEC increased in weaners and does. Bucks had a higher rate of change in FAMACHA in the hot-wet season (*P*<0.0001) as FEC increased. The rate of BCS decline was higher in the post-rainy season in weaners and does (*P*<0.01) and bucks (*P*<0.05) than in other seasons. The decline in PCV was faster during the wet than in the dry seasons. It can be concluded that class and season affected BCS, FAMACHA, and PCV. A linear relationship between FEC and FAMACHA score suggests that FAMACHA could be a good indicator of GIN burden.

## Introduction

The sub-Saharan Africa (SSA) possesses 38 million goats in the Southern African Development Community (SADC) region, where they are kept by resource-limited farmers (Dzama 2016). Goats represent a major asset in resource-limited farmers by providing money through the sale of live goats, meat and hides (Zvinorova et al., 2016). Goats possess the ability to utilize low-quality and undesirable feeds, have minimal input requirements and are easy to manage (Ndlela et al., 2021a). Nguni goats are popular in the SADC region due to the natural selection pressure (Mkwanazi et al., 2020). Their productivity, however, remains marginal due to the high prevalence of parasites and diseases, leading to increased economic losses. Parasite challenges negatively impact goat health. The repercussion of gastrointestinal nematodes (GIN) includes lower fertility and milk production, high veterinary costs, morbidity and mortality (Regassa et al., 2006).

Gastrointestinal nematode burdens change the metabolism of a goat, reducing protein and energy retention and disturb the mineral balance (Rupa and Portugaliza, 2016). Worm burdens are characterized by the loss of body condition, anaemia, diarrhoea and death as significant challenges in goats (Nwoke et al., 2015). Goats survive in hardy conditions with high temperatures, scarce water and feed shortages (Zvinorova et al., 2016). Nevertheless, temperatures are expected to increase due to climate change. Such changes alter the seasonal patterns and increase the number of gastrointestinal parasites (Van Dijk et al., 2010; Morgan and Dijk, 2012). The most pathogenic gastrointestinal parasites of goats commonly encountered include *Haemonchus*, *Strongyloides*, *Trichostrongylus* and *Oesophagostomum* (Mpofu et al., 2020).

The FAMACHA score and PCV are useful tools for predicting anaemia associated with high worm burdens due to the blood-sucking nature of GIN. Hoste et al. (2008) reported an interaction between parasitism and nutrition in goats; however, there is no information on the relationship between parasite loads and the health status of different classes of Nguni goats across seasons. It is, therefore, important to determine the relationship between worm infection and the health status of Nguni goats to establish their adaptability mechanisms and health measures appropriate to detect GIN infection by resource-limited farmers to improve goat health.

Gastrointestinal parasites are sensitive to temperature and moisture as they multiply and proliferate during warm, humid conditions (Ndlela et al., 2021b). Gastrointestinal nematodes were ranked as a major challenge in goat productivity; hence, it is important to relate the prevalence of nematodes to the season and class of goats in the survey. Farmers use several indigenous methods to identify symptoms and disease conditions in goats infected with nematodes, including anaemia, body weight and condition changes. The relationship between faecal egg counts and the health status of different classes of Nguni goats needs to be established to advance indigenous knowledge (IK). Knowing such information is vital for the development of appropriate effective worm control strategies for Nguni goats. Findings from the study may also serve as a reference to other resource-limited areas under similar production systems. The objective of the study was, therefore, to determine the occurrence of GIN infection in different classes of goats and the relationship between faecal egg count (FEC) and health status. The hypothesis tested was that there is no relationship between the faecal egg count and the health status of Nguni goats.

## Materials and methods

### Description of the study site

The study was conducted at Jozini municipality in uMkhanyakude district in the Northern part of KwaZulu-Natal province, lying on 27° 24′ 06.9″ S, 32° 11′ 48.6″ E with altitude ranges of 80 to 1900 m above sea level. Jozini experiences a subtropical climate with an average annual rainfall of 600 mm, occurring mainly in the hot-wet season (December to February). The cool-dry season occurs between June and August (Gush, 2008).

The average daily maximum and minimum temperatures are 20 °C and 10 °C. The vegetation at Jozini consists of coastal sand-veld, bushveld, foothill wooded grasslands (Morgenthal et al., 2006) and poor herbage quality observed through transect walks. Livestock farming is the major livelihood activity. The study was conducted in these randomly selected villages: Nyawushane, Biva, Mkhonjeni, Madonela, Makhonyeni, Mamfene, Mkhayana and Gedleza.

### Goat selection and study design

A list of farmers who kept goats was generated with the assistance of extension officers, chairperson of livestock association and community representatives. Households that had a minimum of five goats for each of three classes were identified. Goats were selected based on the owner’s willingness to participate in the study and the assurance of their availability throughout the study. A total of 120 goats of different class categories and sex were used in the study. Goats were differentiated into three classes of 40 each (weaners (>3 months), does (>1 year), bucks (>1 year)). The class of goats was determined by counting the number of permanent incisors. Does and bucks were ear-tagged, and data were collected from the same goats throughout the experiment.

Weaners could not be marked because they were growing fast to adult stages; therefore, they were randomly selected throughout the year from the same households. Goats were kept under traditional extensive management systems where they grazed on communal rangelands during the day and penned at night throughout the year. Does were not serviced throughout the experiment since they were kept in different camps away from bucks. Veterinary care was low to non-existent, and goats were not dewormed or treated with conventional drugs or ethnoveterinary medicine.

### Data collection

All data were collected on the first day of the study period and then once every season (May 2018 to February 2019). The four seasons of data collection were the cool-dry (August), hot-dry (November), hot-wet (February) and post-rainy (May). Body weight, body condition score, FAMACHA score, packed cell volume and faecal egg count were measured on each goat.

#### Body weight and body condition scoring

The body weights (BW) of goats were estimated using a goat tape developed by the Department of Agriculture in South Africa (De Villiers et al., 2009). The tape was placed around the heart girth, representing the chest’s circumference measured at the most dorsal point of the chest in line with the elbow, bisecting the chest at the approximate position of the heart.

The BCS was determined according to Gerhard et al. (1996) on a scale of 1 to 5, with a score of 1 indicating a thin, emaciated goat and 5 for an obese goat. The BCS was conducted by assessing the amount of fat covering the spine in the loin area, ribs, tailhead and fat pad at the sternum. The BCS was performed by the same two individuals throughout the study to avoid inter-personal discrepancies, and the average scores were considered.

#### FAMACHA scoring

The levels of anaemia were assessed using FAMACHA scores and PCV (Kaplan et al., 2004). The colour of the ocular conjunctiva was scored on a 1–5 scale using a laminated FAMACHA chart that was placed next to the eye of each goat, with 1 (red colour), optimal (indicating a non-anaemic goat); 2 (red-pink), acceptable (non-anaemic); 3 (pink), borderline (mildly anaemic); 4 (pink-white), dangerous (anaemic); and 5 (porcelain white), fatal (a severely anaemic goat).

#### Determination of packed cell volume

Packed cell volume was measured on whole blood samples using the Bull et al. (2003) method. Ethylene diamine tetraacetic acid (EDTA) tubes were used to collect blood samples from each goat through the jugular vein in the morning between 0700 and 0900. Packed cell volume was determined within 6 h of blood collection. Three-quarters of a capillary tube was filled with blood, and one end of the tube was sealed by heating. Capillary tubes were placed in the micro-haematocrit centrifuge, centrifuged at a relative centrifugal force of 2 000 × g for 3 min and read on the haematocrit reader. Results were expressed as the percentage of red blood cells in the total volume of whole blood.

#### Faecal egg counting and identification of nematode larvae

Faecal samples were collected directly from the rectum into ziplock bags. Faecal egg count (FEC) was determined using the McMaster technique (Reinecke, 1973). Faecal pellets were crumpled finely, and 2 g was measured and mixed with 58 ml of 40 % sugar solution. The McMaster slide was filled with the mixture where eggs and oocysts were counted and differentiated directly under the microscope on a 10× magnification. According to Reinecke (1973), faecal cultures were prepared to identify further egg types that were hard to distinguish, where 3 g of faeces was incubated at between 26 and 28 °C for 7 days. The infective larvae were collected using a Baerman Technique. The L_3_ stage nematode larvae were identified according to Van Wyk and Mayhew (2013). Egg count per gramme of faeces was calculated using the formula:

$$EPG=\frac{A\times B}{C\times D\times E\ }$$

where *A* = number of eggs counted, *B* = 60 (total volume of faecal suspension), *C* = number of chambers counted, *D* = grammes of faeces, and *E* = 0.15 ml (standard volume of chamber).

#### Statistical analysis

For the analyses of BCS, FAMACHA score and PCV, PROC UNIVARIATE (SAS, 2012) was used to check data for normality. Logarithmic transformation was applied to the dataset for FEC before analysis. Data were analysed using the general linear model procedure (SAS, 2012) to determine the effect of season and class of goats and their interactions on FEC, BCS, FAMACHA score and PCV. Least square means were compared using the PDIFF procedure (SAS, 2012). Differences among the least square means were considered significant at a confidence interval of 95 %. The model used was as follows:

$${\textrm{Y}}_{\textrm{ij}}=\upmu +{\textrm{M}}_i+{\textrm{L}}_j+{\left(\textrm{M}\times \textrm{L}\right)}_{\textrm{ij}}+{\upbeta}_1\textrm{F}+{\upvarepsilon}_{ij}$$

where:

*Y*_ij_ is the BCS, FAMACHA score and PCV for each goat.

*μ* is the overall mean common to all observations.

*M*_i_ is the effect of the season (hot-wet, post-rainy, cool-dry, hot-dry).

*L*_j_ is the effect of class of the goat (weaner > 3 months old, doe > 1-year-old female, buck > 1-year-old male);

*F* is the effect of faecal egg count (epg).

*β*_1_ *F* is the partial regression coefficient of the dependent variable on FEC.

*ε*_*ij*_ is the residual error. The FEC (epg) was incorporated as a covariate.

For each class of goats, the PROC CORR (SAS, 2012) was used to determine the correlation among FEC, BCS, FAMACHA score and PCV. The PROC REG (SAS, 2012) procedure was used to determine the relationship between FEC and BCS, FAMACHA score and PCV. A *t* test was used to compare the gradients of the graphs after establishing that there were no interactions among class and season.

## Results

### Seasonal distribution of gastrointestinal parasitic infection in goats

**Fig. 1:**
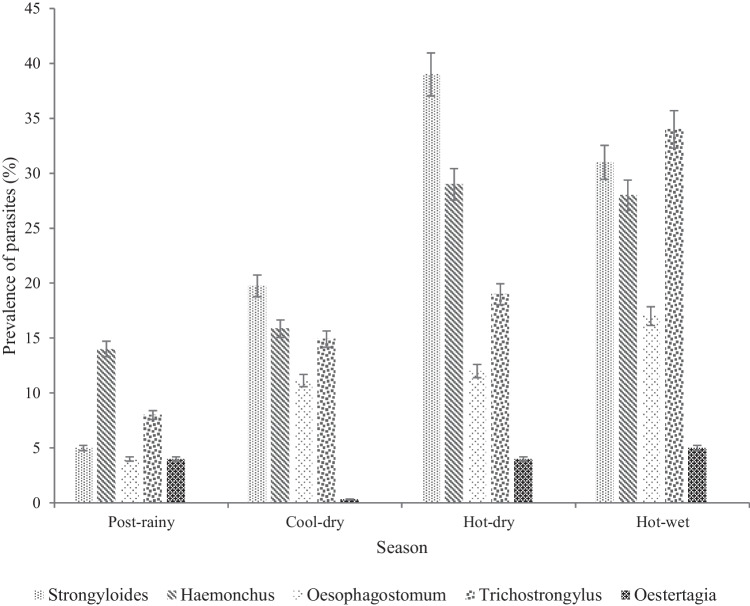
Seasonal occurrence of different species of gastrointestinal nematodes in goats

Figure 1 depicts the distribution of GIN among goats across all seasons. *Strongyloides* egg type had the overall highest prevalence rate, followed by *Haemonchus contortus*, *Trichostrongylus* and *Oesophagostomum* in that descending order. *Ostertagia* had a lower count. *Strongyloides* were highly prevalent during dry seasons, followed by *Haemonchus contortus* and *Trichostrongylus*.

### Body condition score and faecal egg count

Table 1 shows the effects of class of goat and season on FAMACHA, BCS and PCV. There was a significant interaction between season and class of goat on BCS. Class had an effect on BCS (*P*<0.001). The BCS for does and bucks were higher (*P*<0.05) during the hot-wet and post-rainy seasons than the dry seasons. All goat classes had the highest BCS in the post-rainy season. The faecal egg counts, as a covariate, did not affect the BCS of goats.

**Table 1:** Least square means for the season and class on BCS, FAMACHA and PCV of Nguni goats

Class	Season	FAMACHA	BCS	PCV
Weaners	Cool-dry	2.56 ± 0.101^a^	2.83 ± 0.070^b^	25.7 ± 0.79^ab^
	Hot-dry	3.18 ± 0.99^bc^	2.67 ± 0.068^ab^	25.0 ± 0.79^ab^
	Hot-wet	3.12 ± 0.102^bc^	2.67 ± 0.071^ab^	25.1 ± 0.80^ab^
	Post-rainy	2.57 ± 0.99^a^	2.98 ± 0.069^c^	24.6 ± 0.79^a^
Does	Cool-dry	2.69 ± 0.114^a^	2.69 ± 0.079^ab^	26.6 ± 0.90^ab^
	Hot-dry	3.53 ± 0.115^d^	2.47 ± 0.080^a^	25.8 ± 0.91^ab^
	Hot-wet	3.68 ± 0.120^d^	2.83 ± 0.083^b^	26.4 ± 0.94^ab^
	Post-rainy	2.86 ± 0.110^ab^	2.76 ± 0.073^b^	27.4 ± 0.86^c^
Bucks	Cool-dry	2.62 ± 0.124^a^	2.78 ± 0.087^b^	26.6 ± 0.98^bc^
	Hot-dry	3.43 ± 0.134^cd^	2.89 ± 0.093^b^	27.9 ± 1.04^bc^
	Hot-wet	3.52 ± 0.141^d^	2.92 ± 0.092^c^	27.4 ± 1.11^bc^
	Post-rainy	2.76 ± 0.132^a^	3.00 ± 0.091^c^	29.3 ± 1.03^cd^
Levels of significance				
Class		***	***	*
Season		***	**	NS
Class × Season		NS	*	NS

^ab^Within a column, values with different superscripts differ

***P<0.001; ** *P*<0.01; *P<0.05; *NS*, not significant (*P*>0.05)

*BCS*, body condition scoring; *PCV*, packed cell volume; *FEC*, faecal egg count

### FAMACHA score and packed cell volume

There was no interaction between class and season (*P*>0.05) in both FAMACHA and PCV (Table 1). Both FAMACHA scores and PCV were, however, influenced by the class of goats. The season had a significant effect on FAMACHA scores but not on PCV. The FAMACHA scores were lower during the post-rainy season in all goat classes. Weaners had the lowest PCV in the post-rainy season (24.6 ± 0.79), while does and bucks had the highest PCV in that season.

### Correlations and relationships between faecal egg count, FAMACHA score, packed cell volume and body condition score for different classes of goats

Correlation coefficients among BCS, FAMACHA and PCV for the different classes of goats in each season are shown in Table 2. There was a negative correlation between the FAMACHA and BCS in does and weaners across all four seasons. There were no significant correlations between FAMACHA and BCS in bucks. Packed cell volume and BCS were correlated during the dry seasons for weaners but not in the wet seasons. In addition, a weak relationship between FAMACHA and FEC was observed in does and bucks during the hot-dry season. The relationship between PCV and FEC showed contrasting patterns between weaners, does and bucks. In weaners, a significant negative relationship was only observed in the post-rainy season, while for does, the relationship was significant in the hot-dry season. For bucks, the relationship was only observed in the cool-dry season.

**Table 2:** Pearson’s correlation coefficients among BCS, FAMACHA, PCV and FEC of weaners, does and bucks

Season	FAMACHA	Weaners			Does			Bucks	
		PCV	FEC	FAMACHA	PCV	FEC	FAMACHA	PCV	FEC
BCS									
Post-rainy	−0.61**	0.22	−0.33*	−0.38*	0.23	−0.04	−0.10	0.52*	−0.07
Cool-dry	−0.33*	0.32*	−0.13	−0.15	0.30*	−0.17*	−0.03	0.14	−0.08
Hot-dry	−0.28*	0.29*	−0.08	−0.15	0.10	−0.36*	−0.26	0.07	−0.09
Hot-wet	−0.13	0.18	−0.08	−0.17	0.18	−0.17*	−0.04	0.03	−0.02
FAMACHA									
Post-rainy	-	−0.14	0.21	-	−0.11	0.07	-	−0.05	0.10
Cool-dry	-	−0.26	0.14	-	−0.27	0.14	-	−0.22	0.01
Hot-dry	-	−0.35*	0.04	-	−0.24	0.14*	-	−0.34*	0.12**
Hot-wet	-	−0.07	0.02	-	−0.08	0.20	-	0.14	0.08*
PCV									
Post-rainy	-	-	−0.25^*^	-	-	−0.13	-	-	−0.22
Cool-dry	-	-	−0.22	-	-	−0.12	-	-	−0.40*
Hot-dry	-	-	−0.19	-	-	−0.34*	-	-	−0.11
Hot-wet	-	-	−0.14	-	-	−0.17	-	-	−0.24

**P<0.001; *P<0.05; *BCS*, body condition scoring; *PCV*, packed cell volume; *FEC*, faecal egg count

**Fig. 2:**
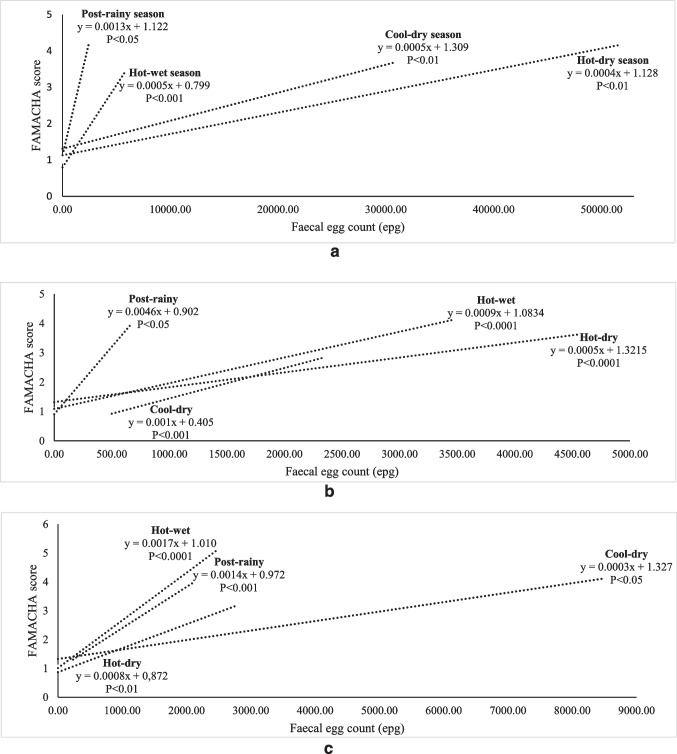
**a** Relationship of seasonal changes between FAMACHA score and faecal egg count in weaners. **b** Relationship of seasonal changes between FAMACHA score and faecal egg count in does. **c** Relationship of seasonal changes between FAMACHA score and faecal egg count in bucks

**Fig. 3:**
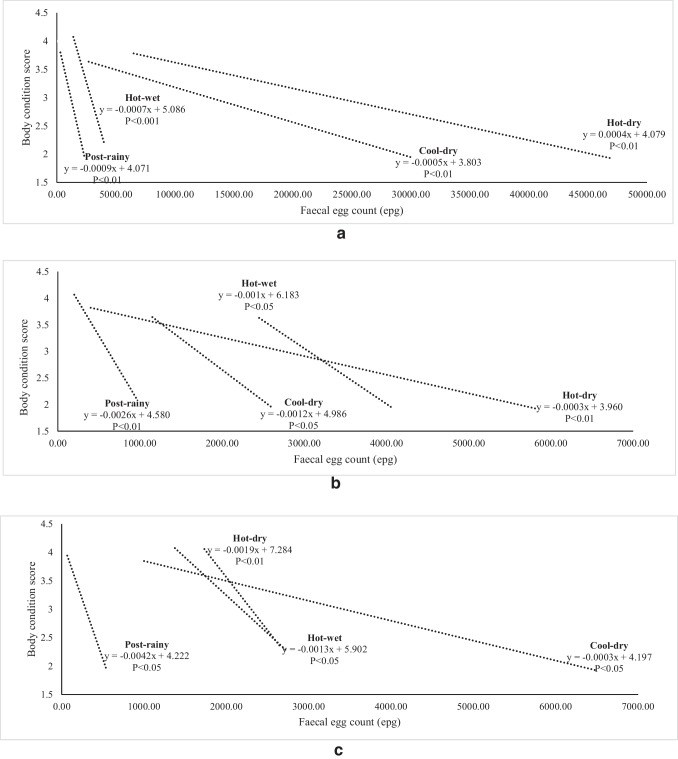
**a** Relationship of seasonal changes between body condition score and faecal egg count in weaners. **b** Relationship of seasonal changes between body condition score and faecal egg count in does. **c** Relationship of seasonal changes between body condition score and faecal egg count in bucks

**Fig. 4:**
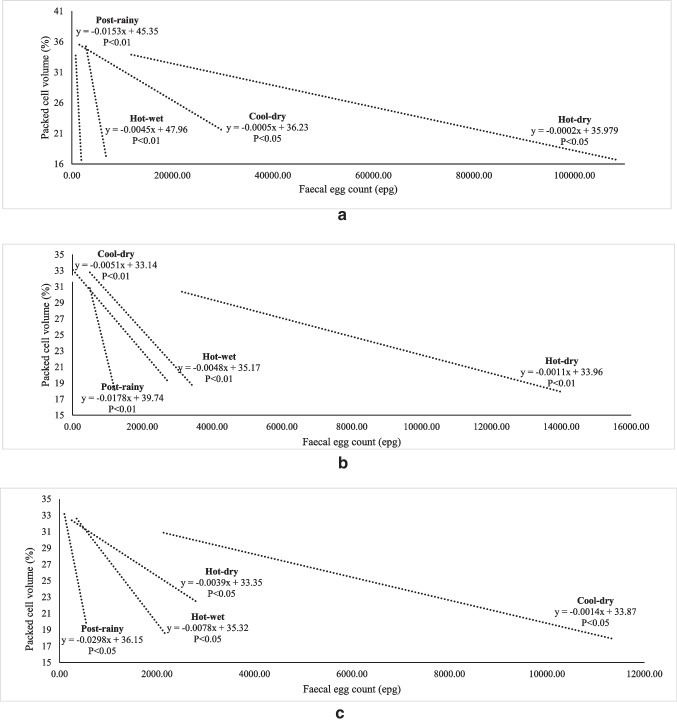
**a** Relationship of seasonal changes between packed cell volume and faecal egg count in weaners. **b** Relationship of seasonal changes between packed cell volume and faecal egg count in does. **c** Relationship of seasonal changes between packed cell volume and faecal egg count in bucks

The relationship between FEC and FAMACHA in weaners, does and bucks is illustrated in Fig. 2a, b and c. All the correlation coefficients were significant. For weaners, the rate of change in FAMACHA score was higher (*P*<0.01) in the hot-wet and post-rainy seasons than in other seasons as FEC increased (*P* < 0.01). In does, the rate of change was similar among the hot-wet and hot-dry seasons. The FEC was generally low in bucks, except in the cool-dry season. As illustrated in Fig. 3, the rate of decline in BCS and FEC was similar among the post-rainy, cool-dry and hot-wet seasons. The hot-dry season showed a slow decline. In weaners, BCS declined sharply during the hot-wet and post-rainy seasons. For bucks, the decline in BCS was low during the cool-dry season. Figure 4 shows the relationship between FEC and PCV in different classes of goats. In weaners and does, the decline in PCV was faster during the wet than the dry seasons (*P*<0.01). In bucks, the rate of change in PCV was higher in all the seasons except the cool-dry season.

## Discussion

Gastrointestinal parasitism is a worldwide challenge affecting goat productivity (Marshall et al., 2012; Emiru et al., 2013; Mpofu et al., 2020). Heavy GIN burden in goats leads to poor health and reduced productivity and increases mortality. Walking long distances during the dry season when vegetation quantity and quality are low has negative effects on the immunity and health status of goats (Mseleku et al., 2020).

The prevalence of strongyles was predominant due to geo-climatic conditions favourable for the development of various species of strongyles nematodes, for example, *Strongyloides* sp., *Trichostrongylus* sp., *Haemonchus contortus* and *Oesophagostomum* sp. The GIN species found in the present study were similar to those reported previously in goats from the study region (Mpofu et al., 2020). The epidemiology of *Ostertagia* sp. is largely unknown in goats in South Africa since they are mostly recognized as cattle parasites. *Ostertagia* sp. infection probably resulted from cross-infection with cattle in communal rangelands as livestock graze together.

The low BCS in weaners during the hot-dry season is attributed to the reduced quality of feed due to lignification of vegetation induced by cold, modifying the structure and chemical components of the cell wall (Mhomga et al., 2012). At times, feed is not available and goats spend long times scavenging for feed. During the hot-wet season, most farmers tether their goats to prevent them from destroying crops. The higher BCS in the post-rainy season could be due to increased feed quality during the rainy season. Goats also have access to crop residues during the cool-dry season.

The finding that fluctuations in BCS are closely linked to seasons agrees with Rumosa Gwaze et al. (2010). In weaners and does, the positive correlation between BCS and PCV, particularly during the cool-dry season indicates that goats with a good condition can control worm burdens due to having higher leucocyte concentrations (Marshall et al., 2012). The observed negative and weak correlation between BCS and worm burden could indicate the resilience of goats to infection under poor quality feed (Mhomga et al., 2012). The resilience of Nguni goats could partly explain the weak negative correlation between worm burden and PCV (Marshall et al., 2012).

The observed low PCV in weaners during the post-rainy season could be attributed to the protein loss and malabsorption from the damaged intestinal mucosa caused by the heavy burden of GIN (Kumar et al., 2015) combined with the reduced feed quality due to increased lignification. There is increased susceptibility of goats to anaemia during feed and water shortages and high worm burden (Rumosa Gwaze et al., 2010; Mseleku et al., 2020). Ironically, does and bucks had the highest PCV values during the post-rainy season. These findings suggest that different classes of goats should be managed differently across seasons. It should be considered that more attention is put on providing iron supplementation to weaned goats so as to improve the growth performance and health status of goats. The observed low PCV in the post-rainy season concurred with the observed low FAMACHA scores.

The increase in FAMACHA with an increase in FEC could be because parasite burdens affect nutrient absorption in goats used by parasites for their growth. The positive correlation between the FAMACHA score and FEC concurs with Marshall et al. (2012). The higher and faster increase of FAMACHA score in weaners as FEC increased in the post-rainy season could be due to the low immune response of growing animals. During the hot-wet season, for example, worm burden is high, and newly weaned goats would not have developed a strong immunity to adapt to the high worm levels that are stimulated by humid conditions. Does and bucks are likely to have adapted to the local conditions. That could partly explain the similar rate of decline in does during the wet and dry seasons. The fast decline in FAMACHA in weaners could be attributed to the increased blood loss and lower levels of erythrocytes caused by higher worm loads, which concurs with Marume et al. (2011). The low FEC in bucks could reinforce the differences among classes of goats in their susceptibility to gastrointestinal nematodes.

Similar results were reported by Mpofu et al. (2020), in which growing goats showed a higher occurrence of parasitic infection than in adults and suckling goats. These findings, however, contradict with Zvinorova et al. (2016). The weaned kids showed maximum egg output because they were exposed to GIN infections during grazing. Contrary, preweaned kids that never grazed do not show symptoms of GIN infection. Weaning stress, change of diet from milk to forage and change of environment from staying within the household to rangelands results in reduced immune system function, contributing towards susceptibility to GIN infection (Magistrelli et al., 2013).

In northern KwaZulu-Natal, kids are usually not allowed to go to communal pastures before they are weaned. Having less opportunity of exposure to nematode infection from communal rangelands, weaners showed higher FEC due to lower immune response levels as compared to older goats. Similar results were obtained in previous reports (Rumosa Gwaze et al., 2010; Zvinorova et al., 2016). Other reports, however, showed no differences between classes of goats on susceptibility to nematodes (Emiru et al., 2013; Nwoke et al., 2015). Gastrointestinal nematode infection could also emanate from poor pen management and cleaning, where parasites might have built up and increased the chances of kids being infected during nursing. Infections could further be exacerbated by overgrazing in infested pastures and the lack of vaccination of kids in resource-limited areas during the weaning stage.

The lower FEC in adult goats could be attributed to repeated natural infections that might have developed immunity of the goats against infection. The positive linear relationship between FEC and FAMACHA demonstrates the usefulness of a FAMACHA chart to identify anaemia in goats. The FAMACHA system could, therefore, be more useful in resource-limited areas as it can also be used by illiterate individuals to identify goats with gastrointestinal parasite burdens requiring anthelmintic treatment from the herd.

## Conclusions

The incidence of parasitic gastrointestinal infection was higher in weaners than does and bucks. Effects of GIN infection were more prominent during the cool-dry season. Class of goats and season contribute to increased FAMACHA score and worm infestations, the decrease in BCS and PCV in Nguni goats. The linear relationships between FAMACHA scores and FEC suggest that FAMACHA could be useful to predict FEC in Nguni goats. Such could demonstrate that resource-limited farmers can save on expenses for laboratory FEC determination. The seasonal patterns of GIN coupled with differences among classes of goats should be considered in devising suitable control strategies for parasitic infections of goats.

## Data Availability

The datasets generated and/or analysed during the current study are not publicly available due to cooperating producer privacy and confidentiality but are available from the corresponding author on reasonable request.
